# Knowledge, practice and associated factors of breast self-examination among female university students of Bangladesh

**DOI:** 10.1016/j.heliyon.2022.e11780

**Published:** 2022-11-21

**Authors:** A.S.M. Ishtiak, Nawshin Ahmed, Foyjunnesa Gaffar, Md. Abdullah Saeed Khan, Ferdousi Yasmeen

**Affiliations:** a National Institute of Preventive and Social Medicine, Dhaka, Bangladesh; b North South University, Dhaka, Bangladesh; c Pioneer Dental College, Dhaka, Bangladesh; d Infectious Disease Hospital, Dhaka, Bangladesh

**Keywords:** Breast self-examination, Knowledge, Practice, Breast cancer

## Abstract

**Background:**

Breast cancer is the most common cancer and leading cause of death worldwide. Breast self-examination (BSE) is a cost-effective tool for self-assessment and for potential early detection of breast cancer in low-resource settings. This study aimed to explore knowledge, practice and associated factors of BSE among female university students of Bangladesh.

**Methods:**

A cross-sectional study was conducted among 400 conveniently selected female students aged ≥18 years from four universities. A pre-tested semi-structured self-administered questionnaire was used for data collection. Univariate and multivariable logistic regression and simple and multiple linear regression analysis was used to assess determinants of knowledge and practice regarding BSE.

**Results:**

A total of 400 students participated from two private and two public universities (100 from each university). The average age of the participants was 20.89 ± 1.72 years (±SD). Of all, 60.5% had presence of knowledge (i.e., heard) about BSE. The average knowledge score was 7.41 ± 3.27 (in a scale of 0–15). Among those who had knowledge about BSE only 10.7% participants practiced it monthly. Being in public university (Adjusted Odds Ratio [aOR]: 3.42, 95% Confidence Interval [CI] 1.73–6.74) and years of education (aOR: 1.42, 95%CI: 1.02–1.97) were significant determinant of presence of knowledge regarding BSE. Moreover, studying in public university (β: 0.99, 95%CI 0.16–1.82) and education years passed (β: 0.51; 95%CI: 0.18–0.85) were associated with a higher knowledge score. Practice of BSE was negatively associated with students’ rural living prior to admission (aOR: 0.26, 95%CI: 0.08–0.79), and positively associated with level of knowledge regarding BSE (aOR: 1.48; 95%CI 0.08–0.79).

**Conclusion:**

This study revealed a general lack of knowledge and practice regarding BSE among female university students. As knowledge and practice of BSE would increase breast cancer awareness and screening acceptance, authorities should address the issue with properly planned strategies.

## 1 Introduction

Breast cancer in women is a major public health burden and the most common cause of cancer death among women in both high and low-resource countries. Recent global cancer statistics indicate that breast cancer incidence is rising at a faster rate in populations of developing regions [1]. Due to lack of a national cancer registry, the incidence of breast cancer in Bangladesh is unknown. However, the Global Cancer Observatory reported an estimated 5-year breast cancer prevalence of 38.35 per 100,000, and 8.3% of the 156,775 new cancer cases in 2020 were due to breast cancer [2].

In developed world, the age-standardized breast cancer mortality dropped by 40% between 1980 and 2020 [3] because of early detection and improved management. In most of the developing countries including Bangladesh patients often present at an advance stage when little or no benefit can be derived from any sorts of therapy [4]. Early diagnosis can be successfully achieved by mass screening either by Mammography, Clinical Breast Examination (CBE) and Breast-Self Examination (BSE) or by the combination of three. Both mammography [5] and CBE [6] were found effective in early detection of breast cancer and, to some extent, reduction of mortality associated with breast cancer. However, a mass screening program using mammography is often impracticable in low-resource settings. In that case, CBE and BSE could be useful alternatives [7]. At present, in a developing country like Bangladesh, it is not a realistic approach to pursue a population-based mass screening program. Hence, CBE were adopted as a screening tool by the Government of Bangladesh [8].

However, BSE could be a useful adjunct to CBE in the detection of susceptible breast cancer mass. Although, the Shanghai [9] study showed that intensive training on BSE does not give mortality benefits in breast cancer, however, it has the advantage of detecting small masses. In addition, BSE might be beneficial for indirectly raising mass awareness about breast cancer and lead to increased acceptance of screening programs based on other methods in low resource settings [10]. Knowledge is essential for behavioral change and obliterating disbelieve and misconception. Thus, knowledge may improve health seeking behavior and enhance screening practice [10]. A systematic review of global guidelines issued after 2010 found that, for average risk women, breast cancer screening through clinical encounter and clinical breast examination is recommended starting from an age of at least 25 years [12]. Till 2003, BSE was a recommended screening tool starting from 20 years of age by American Cancer Society (ACS), it now remains optional for women across all ages [13]. Although, BSE does not provide any mortality benefits against breast cancer, awareness about the procedure could enhance women’s knowledge about the normal appearance and feel of their breasts. Thus, any suspected deviation from normality could be reported early by the suspecting individual. Moreover, many women are comfortable with BSE over CBE as regular screening tool.

In Bangladesh, a study conducted in 2011 found that awareness and practice of BSE among employees of different universities were low [4]. Another study found that in rural areas, breast cancer awareness was found to be riddled with misconceptions and myths [11]. Talking about it was considered shameful and diagnosis of breast cancer might bring devastating consequences in personal lives. Hence, women with breast problems often do not seek healthcare. Therefore, awareness about BSE is expected to help remove misconceptions and empower women regarding their health. In this context, women studying in public and private universities makes a good candidate for exploring awareness about BSE among young educated women. However, the status of knowledge and practice of BSE among female university students of Bangladesh is unknown. Therefore, the objective of this study was to explore the knowledge, practice and associated factors of BSE in a sample of female public and private university students.

## 2 Methods

### 2.1 Study design, setting and population

This cross-sectional descriptive study was conducted among female students of four universities of Dhaka city, Bangladesh between January 2013 to December 2013. The universities included two public (University of Dhaka and Jagannath University) and two private institutes (North South University and American International University). Students aged <18 years, having any physical or mental illness, and unwilling to participate were excluded. As the prevalence of female university students with knowledge on BSE in Bangladesh were unknown, assuming a 50% prevalence, and considering 95% level of significance, 5% error and 5% non-response, the sample size was calculated as 403. However, for convenience a total 400 samples were targeted. Finally, 100 students were purposively selected from each university.

### 2.2 Research instrument

After extensive search of previously published literature and discussion with experts, a semi-structured self-administered questionnaire was developed for data collection. It was piloted in 30 randomly selected female students of a private university in order to clarify any ambiguity in wording, and to ensure an appropriate length of the questionnaire and adequacy of questions. The question patterns were adjusted based results of piloting and feedback of experts. The final version consisted of four parts- Sociodemographic variables and family history of breast cancer, sources of information regarding BSE, knowledge about BSE and practice of BSE. The sociodemographic part asked about participant’s age, university name, current year of education, religion, marital status, residence, parent’s educational status and occupation, family size, monthly family income, and family history of cancer. Participants were queried if they have heard about BSE and, if yes, from where they got the information, in the second part of the questionnaire. In the final part, the knowledge and self-reported practice of BSE was assessed by a set of twelve and four questions, respectively (See Supplementary file 1 for the detailed questionnaire).

### 2.3 Data collection procedure

At first colleagues and faculty members of these universities were contacted for collaboration or assistance in data collection. Then the questionnaire was distributed to female students through respective faculty members of different universities. Students were conveniently sampled from classes.

### 2.4 Scoring scheme

At first, to assess the knowledge of participants regarding BSE a question was asked to know if they have heard the term ‘Breast Self-Examination’ previously. An affirmative answer to this question was regarded as positive for presence of knowledge regarded BSE. Then participants having knowledge of BSE was asked a set of 12 questions related to BSE. For these questions, each correct answer was assigned a score of 1, while an incorrect answer or “do not know” was awarded a score of 0. As two items had more than one correct answer (one with two and another with three correct answers), the maximum score for 12 knowledge related questions equated to 15 (Check supplementary file 1 for the scoring scheme). A total score for each participant was computed by summing the number of correct answers.

### 2.5 Statistical analysis

Descriptive and analytic statistic was carried out to describe the findings of this study. Categorical data was presented as frequency (percentage) and continuous data was presented as mean ± SD. Chi-square test and Fisher's exact test was used to determine association between categorical variables, where appropriate. While independent samples t test was used for continuous data. Univariate and multivariable logistic regression analysis was used to determine factors associated with presence of knowledge and practice of BSE among participants. Simple and multiple linear regression analysis was used to assess factors associated with level of knowledge. Independent variables for multivariable logistic regression and multiple linear regression were selected based on p value (<0.05) of the univariate analysis. However, for multiple linear regression age was excluded due to collinearity with years of education. Statistical software SPSS (version 24) was used for analysis. The reliability statistic Cronbach’s alpha (α) for the knowledge portion of the questionnaire was 0.68 for our sample.

### 2.6 Ethical measures

The study was approved by the Ethical Review Board of National Institute of Preventive and Social Medicine (Memo No. NIPSOM/EC/2013/07–10). All procedures were conducted in accordance with the Declaration of Helsinki. Informed written consent was taken from the participants before inclusion in the study.

## 3 Result

The mean age of participants was 20.9 ± 1.7 years (±SD). Majority (30%) were at their 2^nd^ undergraduate years. Among all, 7.8% were married, 34.3% were living in hostel, and 22% came from rural area. Majority students' fathers had postgraduate education (42.1%) and mothers had secondary/higher secondary education (50.4%). Most of the students (34.0%) had a monthly family income of 10001–30000 BDT and >4 family members (53.5%). Private and public university students did not have any significant difference in relation to age, educational years, and marriage (p > 0.05). However, a significantly higher proportion of public university students were living in hostels (p < 0.001), came from rural areas (p < 0.001) and had >4 family members (<0.001) compared to that of private university students. On the other hand, a significantly higher proportion of private university students’ fathers and mothers had post-graduation (p < 0.001 for both), and had a monthly family income >60000 BDT (p < 0.001) compared to their counterparts. Among all students, 15.5% had family history of breast cancer and the proportion was significantly higher among those studying in private universities (p < 0.001). A total of 60.5% participants had knowledge (i.e., heard) about BSE with the proportion being significantly higher in public university students (75%) than private university students (46%; p < 0.001). The average knowledge score of all students was 7.4 ± 3.3. Again, it was significantly higher in public university students (7.8 ± 3.6) than students from private universities (6.8 ± 2.7; p < 0.001). Among those who had knowledge, 20.7% students practiced BSE at least once a year. However, no difference were noted between students of public and private universities in relation to practice. Notably, only 10.7% participants with knowledge of BSE practiced it monthly (Table 1).

**Table 1:** Sociodemographic profile, knowledge and practice of breast self-examination among participants.

Variable	Total n (%)	Private University n (%)	Public University n (%)	p-value
**Age (years)**	20.89 ± 1.72	20.76 ± 1.71	21.02 ± 1.72	0.124
**Education**				
Undergraduate 1^st^ years	119 (29.8)	64 (32.0)	55 (27.5)	0.812
Undergraduate 2^nd^ year	120 (30.0)	56 (28.0)	64 (32.0)	
Undergraduate 3^rd^ year	80 (20.0)	39 (19.5)	41 (20.5)	
Undergraduate 4^th^ year	59 (14.8)	31 (15.5)	28 (14.0)	
Graduate	22 (5.5)	10 (5.0)	2 (6.0)	
**Marital status**				
Unmarried	369 (92.3)	185 (92.5)	184 (92.0)	1.000
Married	31 (7.8)	15 (7.5)	16 (8.0)	
**Current residence**				
Living with family	263 (65.8)	181 (90.5)	82 (41.0)	<0.001
Living in hostel	137 (34.3)	19 (9.5)	118 (59.0)	
**Residence before admission**				
Urban	312 (78.0)	179 (89.5)	133 (66.5)	<0.001
Rural	88 (22.0)	21 (10.5)	67 (33.5)	
**Father’s education**∗				
Primary	2 (0.5)	0 (0.0)	2 (1.0)	<0.001
Secondary and higher secondary	73 (18.9)	14 (7.3)	59 (30.1)	
Graduate	149 (38.5)	66 (34.6)	83 (42.3)	
Post-graduate	163 (42.1)	111 (58.1)	52 (26.5)	
**Mother’s education**∗				
Primary	7 (1.9)	0	7 (3.6)	<0.001
Secondary/higher secondary	189 (50.4)	59 (33.0)	130 (66.3)	
Graduate	134 (35.7)	88 (49.2)	46 (23.5)	
Post-graduate and above	45 (12.0)	32 (17.9)	13 (6.6)	
**Monthly family income (BDT)**∗				
≤10000	22 (5.8)	1 (0.5)	21 (11.1)	<0.001
10001–30000	128 (34.0)	8 (4.3)	120 (63.5)	
30001–60000	121 (32.1)	85 (45.2)	36 (19.0)	
>60000	106 (28.1)	94 (50.0)	36 (19.0)	
**Number of family members**				
≤4	186 (46.5)	113 (56.5)	73 (36.5)	<0.001
>4	214 (53.5)	87 (43.5)	127 (63.5)	
**Family History of Breast Cancer**				
Present	62 (15.5)	50 (25.0)	12 (6.0)	<0.001
Absent	338 (84.5)	150 (75.0)	188 (94.0)	
**Knowledge about BSE**				
Present	242 (60.5)	92 (46.0)	150 (75.0)	<0.001
Absent	108 (54.0)	50 (25.0)	158 (39.5)	
**Knowledge level**∗∗	7.41 ± 3.27	6.79 ± 2.68	7.79 ± 3.55	0.014
**Practice about BSE**∗∗				
Never	192 (79.3)	71 (77.2)	121 (80.7)	0.624
At least once a year	50 (20.7)	21 (22.8)	29 (19.3)	
Once a month	18 (7.4)	2 (2.2)	16 (10.7)	

BSE: Breast self-examination.

∗ Excluding missing values.

∗∗ Among those who knows about breast self-examination. Knowledge level is measured on a scale of 1–15.

Univariate logistic regression analysis found that age, university type, education years, current residence, father’s education, monthly family income and number of family members were significantly associated with presence of knowledge about BSE. However, multivariable logistic regression revealed that after adjustment of those factors, students of public university were 3.4 times (95%CI: 1.7–6.7) more likely to have knowledge of BSE than that of private university. Additionally, seniority of a year in the university was associated with 1.4 times (95%CI: 1.0–2.0) higher chance of getting knowledge of BSE (Table 2).

**Table 2:** Logistic regression analysis of factors associated with presence of knowledge about breast self-examination among participants.

Variable	Crude OR (95%CI)	*p*-value	Adjusted OR (95%CI)	*p-*value
**Age (years)**	1.39 (1.22–1.58)	**<0.001**	1.12 (0.90–1.41)	0.312
**University type**				
Private	Ref		Ref	
Public	3.52 (2.31–5.38)	**<0.001**	3.42 (1.73–6.74)	**<0.001**
**Education (years)**	1.65 (1.37–1.99)	**<0.001**	1.42 (1.02–1.97)	**0.036**
**Marital status**				
Unmarried	Ref			
Married	1.66 (0.74–3.70)	0.218		
**Current residence**				
Living with family	Ref		Ref	
Living in hostel	2.08 (1.33–3.24)	**0.001**	1.12 (0.61–2.09)	0.713
**Residence before admission**				
Urban	Ref		Ref	
Rural	1.64 (0.99–2.71)	**0.057**	1.09 (0.57–2.10)	0.793
**Father’s education**				
Up to higher secondary	Ref		Ref	
Graduation and above	0.57 (0.33–0.98)	**0.044**	1.05 (0.54–2.04)	0.940
**Mother’s education**				
Up to higher secondary	Ref			
Graduation and above	0.81 (0.53–1.23)	0.320		
**Monthly family income (BDT)**				
>45000	Ref		Ref	
≤45000	2.11 (1.38–3.22)	**0.001**	0.81 (0.42–1.56)	0.534
**Number of family members**				
≤4	Ref		Ref	
>4	1.70 (1.13–2.54)	**0.010**	1.33 (0.83–2.14)	0.237
**Family history of breast cancer**				
Absent	Ref			
Present	1.12 (0.70–2.15)	0.482		

To assess the factors associated with level of knowledge about BSE among students who knew about it, simple and multiple linear regression analyses were carried out. In the simple linear regression, age, university type, and education years came as significant determinants. In the multiple linear regression, only university type and education were put as regressors. Because, age and education years were correlated. The analysis found that being in the public university was associated with 1.0 points (95%CI: 0.2–1.8) increase in knowledge score. Moreover, one year passage in the university was associated with a higher score (β: 0.5; 95%CI: 0.2–0.9) in knowledge of BSE (Table 3).

**Table 3:** Linear regression analysis of factors associated with level of knowledge about breast self-examination among participants.

Variable	Crude OR (95%CI)	*p*-value	Adjusted OR (95%CI)	*p-*value
**Age (years)**	0.37 (0.09–0.59)	**0.009**		
**University type**				
Private	Ref		Ref	
Public	1.00 (0.154–1.85)	**0.021**	0.99 (0.16–1.82)	**0.020**
**Education (years)**	0.52 (0.18–0.86)	**0.003**	0.51 (0.18–0.85)	**0.003**
**Marital status**				
Unmarried	Ref			
Married	-0.26 (-1.70–1.19)	0.729		
**Current residence**				
Living with family	Ref			
Living in hostel	0.35 (-0.49–1.20)			
**Residence before admission**				
Urban	Ref			
Rural	-0.57 (-1.53–0.38)	0.237		
**Father’s education**				
Up to higher secondary	Ref			
Graduation and above	0.27 (-0.73–1.26)	0.597		
**Mother’s education**				
Up to higher secondary	Ref			
Graduation and above	-0.07 (-0.91–0.78)	0.875		
**Monthly family income (BDT)**				
>45000	Ref			
≤45000	-0.65 (-1.49–0.20)	0.135		
**Number of family members**				
≤4	Ref			
>4	-0.18 (-0.43–0.67)	0.671		
**Family history of breast cancer**				
Absent	Ref			
Present	-0.14 (-1.25–0.98)	0.811		

Table 4 describes the results of univariate and multivariable logistic regression of factors associated with practice of BSE among students who had knowledge about the procedure. Age, education years, residence before admission, father’s education, number of family members and knowledge score came as significant determinants in the univariate analysis. However, multivariable analyses found that students who got admitted in the university from rural areas were 74% less likely (95%CI: 0.1–0.8) to practice BSE than those who came from urban areas. Also, one unit increase in knowledge score was associated with 1.5 time (95%CI 1.3–1.7) higher chance of BSE practice among students.

**Table 4:** Logistic regression analysis of factors associated with practice of breast self-examination among participants who had knowledge about it.

Variable	Crude OR (95%CI)	*p*-value	Adjusted OR (95%CI)	*p-*value
**Age (years)**	1.25 (1.03–1.53)	**0.024**	1.22 (0.85–1.76)	0.283
**University type**				
Private	Ref			
Public	0.81 (0.43–1.53)	0.515		
**Education (years)**	1.29 (0.99–1.66)	**0.054**	0.86 (0.54–1.37)	0.513
**Marital status**				
Unmarried	Ref			
Married	0.358 (0.08–1.58)	0.177		
**Current residence**				
Living with family	Ref			
Living in hostel	1.33 (0.71–2.48)	0.374		
**Residence before admission**				
Urban	Ref			
Rural	0.34 (0.14–0.84)	**0.020**	0.26 (0.08–0.79)	**0.018**
**Father’s education**				
Up to higher secondary	Ref			
Graduation and above	1.77 (1.02–3.07)	**0.044**	0.64 (0.25–1.66)	0.356
**Mother’s education**				
Up to higher secondary	Ref			
Graduation and above	0.85 (0.45–1.60)	0.612	0.60 (0.29–1.23)	0.163
**Monthly family income (BDT)**				
>45000	Ref			
≤45000	1.13 (0.60–2.14)	0.709		
**Number of family members**				
≤4	Ref		Ref	
>4	0.52 (0.28–0.98)	**0.043**	0.60 (0.28–1.23)	0.163
**Family history of breast cancer**				
Absent	Ref			
Present	1.85 (0.86–3.96)	0.114		
**Knowledge score**	1.45 (1.28–1.65)	**<0.001**	1.48 (1.29–1.70)	**<0.001**

## 4 Discussion

Early detection and management of breast cancer through screening is crucial. Among the screening methods, Breast self-examination (BSE) is a cost-effective and suitable one for low- and middle-income countries. However, practice of BSE remains low among women of Bangladesh [14]. Therefore, an exploration of the knowledge, and practice of BSE among female university students was carried out.

Our study found that three-fifth of the participants heard about the term BSE with a mean knowledge score of 7.4 ± .3.3 (out of 15). In contrast, some recent studies conducted among female university students of Ethiopia and Eritrea [15, 16, 17] found knowledge of BSE among less than one-third of the participants. On the other hand, another study from Gaza found that nearly all (96.5%) have heard about BSE [18]. But a clear regional variation exists in BSE awareness which was described in detail in a large study covering 24 low, middle-income and emerging economy countries by Pengpid and Peltzer [14]. Their study revealed that the overall BSE awareness was 50.4% in university students, with the proportion being lower than 50% in majority Sub-Saharan and South Asian countries. However, the presence of knowledge (or, awareness) might be affected by how it was defined in the study design. Pengpid and Peltzer asked their participants if they knew about breast self-examination. Knowledge was considered present for a positive answer [14]. Our study adopted the same approach to assess the ‘presence of knowledge’ regarding BSE among the participants. Moreover, we explored the ‘level of knowledge’ through a set of 12 questions. The correct answers were a given a score of one. However, there could be disagreement on what constituted a correct answer for a few questions. For example, we considered age 20 as the correct starting age of BSE based on available texts [19]. However, BSE by the women at their twenties often leads to detection of palpable fibroadenoma which is more common at this age [20]. This often leads to benign biopsies [9] and increased frustration. Hence, an older age could be considered more appropriate starting point for BSE. But considering the awareness impetus of BSE and based on recent studies [21, 22] age 20 could also be considered as the correct starting age. Another important point to be noted is that the Shanghai study [9] showed that there is no preferred method of BSE which could bring survival benefits in breast cancer patients. Hence, the knowledge portion of our questionnaire mainly contained the general points of any self-examination procedures relevant for breast cancer irrespective of BSE methods.

Knowledge about BSE can be affected by many different factors. We found that year of education and university types (private or public) were significant determinants of presence of knowledge as well as level of knowledge among students. An association of education with presence of knowledge about BSE were observed in many studies in different groups of women including those attending health care facilities [23], rural and urban community dwellers [24, 25], and employees [26]. We noted that one year increase in education was associated with 0.51 unit increase in knowledge score. As with each year of education students come in contact with more and more people and information sources, a raise in awareness with time could be explained. As meeting more people increases the likelihood of discussing the topic with someone, this could impart good amount of knowledge regarding BSE. Mihret *et al* observed similar findings among female university students [15]. Interestingly, this could also explain why public university students were more likely to have heard about BSE in our study. We observed that public university students predominantly stayed in the hostels compared to private university students who stayed at their home. In hostels, students are more likely to discuss various topics with their peers, particularly, regarding topics like BSE which is often considered shameful [11]. Consequently, students staying at hostels gain more knowledge about BSE from their surroundings.

We noted that, nearly four-fifth of our participants who had knowledge didn’t practice BSE ever which is higher than that found later by Pengpid and Peltzer [14]. However, our finding is concordant to that of India, China, Singapore, and South Africa [14]. In contrast, Rasu *et al*, in a study conducted in Bangladesh, found that 59% of the university employees who were aware about BSE practiced it [4] and the proportion was significantly higher among more educated participants. We also found that knowledge about BSE significantly determines practice of BSE. Such associations were described in many similar studies [17, 24, 26, 27, 28, 29, 30, 31]. Unlike Mihret *et al* [15] and Alam *et al* [2] we didn’t find any association of family history of breast cancer with BSE practice. But we noted that students originally coming from rural areas were less likely to practice BSE than others. The reluctance may stem from experiences of women in the community. Story *et al* [11] while attempting to recruit participants for a clinical trial relevant to breast cancer in Bangladesh in 2012, found that the consequence of diagnosing with breast cancer might be devastating for the women as they might face rejection, divorce or even a stimulus for suicide. All these clearly outlines the importance of raising awareness and removing stigma surrounding breast cancer in the country.

Although our study is one of the fewer attempts to explore knowledge and practice of BSE among university students, it was not without limitations. The major limitation of the study is the target population. We acknowledge that the ideal target group would have been older women with little or no education. However, university students were chosen for convenience and get an initial idea about the status of awareness on BSE among younger educated women. Another limitation was convenience (non-random) sampling method rendering the study findings non-generalizable to the target population. To get around the problem and make it as generalizable as possible, we purposively choose two public and two private universities so that the sample reflects all socioeconomic strata in our sample. In addition, we selected students from different departments of the universities so that none is missed. Still, the study comprised a small sample and covered only four universities. A qualitative assessment of factors determining practice of BSE could not be done, potentially leaving important determinants. But we recommend further large sample mixed method studies to find the current state of knowledge and practice and associated factors among susceptible women.

## 5 Conclusion

The present study revealed a moderate knowledge and low practice about breast self-examination among female university students. Years of education and university type were discovered to be significant determinants of knowledge and practice. Respective authorities should plan and execute awareness programs to increase breast self-examination knowledge and practice with an aim to increase breast cancer awareness and screening acceptance among women, and to empower them regarding their health.

## Declarations

### Author contribution statement

ASM Ishtiak: Conceived and designed the experiments; Performed the experiments; Contributed reagents, materials, analysis tools or data.

Nawshin Ahmed: Analyzed and interpreted the data; Contributed reagents, materials, analysis tools or data.

Foyjunnesa Gaffar; Ferdousi Yasmeen: Conceived and designed the experiments; Contributed reagents, materials, analysis tools or data.

Md. Abdullah Saeed Khan: Analyzed and interpreted the data; Wrote the paper.

### Funding statement

This research did not receive any specific grant from funding agencies in the public, commercial, or not-for-profit sectors.

### Data availability statement

Data will be made available on request.

### Declaration of interest’s statement

The authors declare no conflict of interest.

### Additional information

No additional information is available for this paper.

## Appendix A Supplementary data

Supplementary File 1 revised Supplementary File 1 revised
