# Supplementary material for: Knowledge, practice and associated factors of breast self-examination among female university students of Bangladesh
Source: Heliyon. 2022 Nov 21;8(11):e11780. doi: 10.1016/j.heliyon.2022.e11780 (PMC9699960; doi:10.1016/j.heliyon.2022.e11780)
Supplement: Supplementary File 1 revised [file mmc1.docx]

**Title of the study**: **Knowledge, practice and associated factors of breast self-examination among female university students of Bangladesh**

Registration no. :

***Sociodemographic information and family history of breast cancer***

1| Age :

2| Name of the University :

3| Current educational status : 1^st^ year 2^nd^ year 3^rd^ year

321

21

1

4^th^ year Masters

54321

4321

321

21

1

4| Religion: Islam Hindu Christian

54321

Buddhist Others………….

4321

5| Marital status: Unmarried Married

1

21

Divorced Others…………..

4321

321

6| Present residential status: Living with family

1

Living in hostel

21

7| Previous residential status: Urban Rural

1

21

8| Father's occupation: Workless Business

21

1

Service Others………...

321

4321

9| Mother's occupation: Housewife Business

4321

321

21

1

Service Others………...

10| Father's educational status :

11| Mother's educational status :

12| Number of family member :

13| Monthly family income : Taka

14| Do you have any family history of cancer? Yes No

121

2121

***Sources of information regarding breast self-examination***

15| Have you heard about Breast Self-Examination? Yes No

121

2121

16| If yes from where you heard about BSE (Breast Self-Examination)?

Health personnel Family Friends

321

1

21

Television Radio Internet

654321

54321

4321

Seminar Newspaper/books/ magazines Others ........

9

87654321

7654321

***Knowledge about breast self-examination***

17| Why Breast Self-Examination should be performed?

As regular check-up If suffering from breast problem

121

2121

Do not know

321

18| What can be detected through BSE?

121

Only breast cancer Any breast problem Do not know

321

2121

19| At which age BSE (Breast Self-Examination) should begin?

At the age of 20 At the age of 30 At the age of 40

2321

1

34321

At the age of 50 Do not know

434321

5434321

20| How often BSE should be performed?

Daily Weekly Monthly

321

21

1

Annually Do not know

54321

4321

21| Up to which age BSE should be performed?

Up to 40 years Up to 50 years Up to 60 years

321

1

21

Throughout the life Do not know

4321

54321

22| In which posture, inspection during BSE should be done?

Standing any where Standing in front of mirror

21

1

Lying Do not know

4321

321

23| What should be checked during inspection? (could be more than one correct answer)

Change in the size and shape of the breast

1

Discoloration or dimpling of the breast Do not know

321

21

24| What should be checked during palpation? (could be more than one correct answer)

Pain in the breast Lump in the breast or under armpit

1

21

Discharge from the nipple Do not know

321

41

25| Which hand should be used to perform BSE?

Same hand Contra lateral hand Do not know

21876541

321

1876541

26| How BSE should be performed?

Palpate with pad of the fingers Palpate with palm

21876541

1876541

Do not know

321876541

27| Which breast should be examined during BSE?

Randomly one breast Both breast Do not know

21876541

321876541

1876541

28| Which area should be covered during BSE?

Breast only Breast and armpit

1876541

21876541

Whole breast up to collar bone, armpit, area between breast

321876541

and armpit Do not know

41

***Practice of breast self examination***

29| Have you performed BSE before?

Yes No

1876541

21876541

30| How frequently do you perform BSE?

Once a month Once in 2 months

21

1

Once in 3 to 5 months Twice a year Once a year

4321

54321

321

31| At what age did you start BSE?

32| When was the last time you performed BSE?

Last month 3-6 months ago

21

1

1year ago Others......................

321

4321

**Answer keys**

Keys to correct answers of BSE knowledge related questions

| Question no | Correct Answer(s) |
| --- | --- |
| 17 | 1 |
| 18 | 2 |
| 19 | 1 |
| 20 | 3 |
| 21 | 4 |
| 22 | 2 |
| 23 | 1, 2 |
| 24 | 1, 2, 3 |
| 25 | 2 |
| 26 | 1 |
| 27 | 2 |
| 28 | 3 |
